# Asia-Pacific venous thromboembolism consensus in knee and hip arthroplasty and hip fracture surgery: Part 2. Mechanical venous thromboembolism prophylaxis

**DOI:** 10.1186/s43019-021-00101-7

**Published:** 2021-06-30

**Authors:** Chavarin Amarase, Aree Tanavalee, Viroj Larbpaiboonpong, Myung Chul Lee, Ross W. Crawford, Masaaki Matsubara, Yixin Zhou, Asia-Pacific (AP) Region Venous Thromboembolism (VTE) Consensus Group, Aasis Unnanuntana, Alvin Tan, Anthony Pohl, Apisak Angsugomutkul, Apisit Patamarat, Arak Limtrakul, Azhar Merican, Azlina Abbas, Badrul Shah Badaruddin, Boonchana Pongcharoen, Bui Hong Thien Khanh, Cao Li, Chaithavat Ngarmukos, Charlee Sumettavanich, Chavanont Sumanasrethakul, Chee-Ken Chan, Cheng-Fong Chen, Chong Bum Chang, Chotetawan Tanavalee, Christopher Scott Mow, Chumroonkiet Leelasestaporn, Chun Hoi Yan, Dang-Khoa Tran, David Campbell, David Liu, Edi Mustamsir, Edsel Fernandez Arandia, Eun Kyoo Song, G. Ruslan Nazaruddin Simanjuntak, Hirotsugu Muratsu, Hyonmin Choe, Jamal Azmi Mohammad, Jason Chi Ho Fan, Ji Hoon Bae, Ji-Wan Kim, Jose Antonio San Juan, Jose Fernando C. Syquia, Jun-Ho Kim, Kang-Il Kim, Ki Ki Novito, Kriskamol Sithitool, Manoon Sakdinakiattikoon, Mel S. Lee, Mohamad Zaim Chilmi, Myint Thaung, Narathorn Kongsakpaisal, Ngai Nung Lo, Nicolaas Budhiparama, Nikom Noree, Nobuhiko Sugano, Paphon Sa-ngasoongsong, Pariwat Taweekitikul, Peter Bernardo, Piti Rattanaprichavej, Piya Pinsornsak, Po-Kuei Wu, Pongsak Yuktanandana, Pruk Chaiyakit, Rahat Jarayabhand, Rami Maher Sorial, Ryuji Nagamine, Saradej Khuangsirikul, Saran Tantavisut, Satit Thiengwittayaporn, Seng Jin Yeo, Siwadol Wongsak, Srihatach Ngarmukos, Sukit Saengnipanthkul, Supparurk Suksumran, Surapoj Meknavin, Thakrit Chompoosang, Than Win, Thana Narinsorasak, Thana Turajane, Thanainit Chotanaphuti, Thanarat Reancharoen, Tokifumi Majima, Ukrit Chaweewannakorn, Viroj Kawinwonggowit, Wanshou Guo, Weerachai Kosuwon, Wei Chai, William J. Maloney, Yee Hong Teo, Yunsu Chen, Yutaka Inaba, Yutthana Khanasuk

**Affiliations:** 1 Department of Orthopaedics, Faculty of Medicine, King Chulalongkorn Memorial Hospital, Chulalongkorn University, Bangkok, Thailand; 2 Orthopedics Department, Police General Hospital, Bangkok, Thailand; 3 Department of Orthopedic Surgery, Seoul National University Hospital, Seoul, South Korea; 4 Orthopaedic Research Unit, Queensland University of Technology, Brisbane, Australia; 5 epartment of Orthopaedic Surgery, Nissan Tamagawa Hospital, Tokyo, Japan; 6 Department of Orthopaedic Surgery, Beijing Jishuitan Hospital, Fourth Clinical College of Peking University, Beijing, China

## Statements of Group 2: Mechanical Venous Thromboembolism Prophylaxis

### 1. Which devices have evidence supporting effective mechanical venous thromboembolism (VTE) prophylaxis in knee and hip arthroplasty and hip fracture surgery?

#### Recommendation

Mechanical devices that have evidence support as an effective mechanical VTE prophylaxis include intermittent pneumatic compression devices (IPCDs), venous foot pump (VFP) devices, and graduated compression stockings (GCSs).

Delegate vote: Agree 94.5%, Disagree 4.1%, Abstain 1.4% (Strong Consensus)

#### Justification

According to the literature, many studies have demonstrated that mechanical devices provide effectiveness as a VTE prophylaxis [1–4]. A systematic review compared the use of an IPCD and an anticoagulant for prophylaxis of VTE events (14 randomized controlled trials [RCTs] and three observation studies) in patients undergoing joint arthroplasty [3]. VTE events occurred in 163 patients (11%); however, there was no statistically significant difference between the IPCD group and the anticoagulation group in VTE events [3]. Subgroup analyses separately evaluating IPCD alone vs. anticoagulation and IPCD plus anticoagulation vs. anticoagulation alone suggested that the combination of IPCD plus anticoagulation may provide a substantial protective effect against VTE events [3]. However, a retrospective study on 1259 primary total knee arthroplasties (TKAs) in a Korean population reported that the use of an IPCD alone did not reduce the DVT incidence in ethnic groups with low DVT incidence [5].

A VFP is a variation of IPCD that performs intermittent pumping only on the foot [4]. The proper length of IPCD to reduce the incidence of VTE is controversial. A meta-analysis comparing the effectiveness of different IPCDs in the prevention of VTE in patients after total hip arthroplasty (THA) found that only one study had fulfilled the eligibility criteria for inclusion in this systematic review [4]. That study enrolled 121 patients and assessed thrombogenesis using the D-dimer level before and after THA for VTE diagnosis [2]. Evaluation for postoperative swelling by measuring the thigh and lower leg circumference was investigated [2]. Fifty-eight patients were assigned to the calf-thigh pneumatic compression group, and the other 63 were assigned to the plantar compression group [4]. At seven days postoperatively, the mean D-dimer levels of the calf-thigh compression and the plantar compression groups were not statistically different (8.86 and 9.26 μg/ml, respectively) [2]. However, there was a significant difference in increasing of the circumference of the thigh after hip arthroplasty, with an averaged 1.22% increase in the calf-thigh compression group and 3.19% increase in the plantar compression group [2]. Calf-thigh pneumatic compression was more effective than plantar compression for reducing thigh swelling during the early postoperative stage [2]. At 3 weeks after THA, there were no patients with symptomatic deep vein thrombosis (DVT) or pulmonary embolism (PE) in either the calf-thigh compression or the plantar compression group [2]. Also, another RCT trial from Japan reported that the VFP provided a significantly lower rate of PE as compared to results for the control group [1].

Regarding a meta-analysis study on GCSs for the prevention of DVT, 19 RCTs were identified involving 1681 patients and 1064 legs for a total of 2745 analytic units [6]. Nine studies included patients undergoing general surgery, six studies included patients undergoing orthopedic lower limb surgery, and one study included medical patients [6]. In the treatment group, the GCS was applied from patient admission until they were fully mobile or discharged. In the treatment group (GCS), 9% of patients developed DVT (126 of 1391 units) in comparison with 21% in the control group (282 of 1354 units) [6]. An overall effect of VTE prophylaxis favored treatment with a GCS (*p* < 0.00001) [6].

### 2. Do all mechanical devices (IPCD, VFP, and GCS) provide similar efficacy for VTE prophylaxis?

#### Recommendation

Inconclusive, there are not enough supporting evidences.

Delegate vote: Agree 95.9%, Disagree 1.4%, Abstain 2.7% (Strong Consensus)

#### Justification

There has been a lack of strong evidence that directly compares the effectiveness of the different mechanical devices (IPCD, VFP, and GCS) in VTE prophylaxis. However, most experts agree that all mechanical devices for VTE prophylaxis in knee and hip arthroplasty and hip fracture surgery do not provide similar effectiveness.

### 3. Besides a mechanical device, what other nonpharmacological methods can enhance VTE prophylaxis in knee and hip arthroplasty and hip fracture surgery?

#### Recommendation

Early ambulation and leg elevation can be added in the postoperative protocol of knee and hip arthroplasty and hip fracture surgery with a tendency to provide a positive effect on mechanical VTE prophylaxis.

Delegate vote: Agree 97.2%, Disagree 1.4%, Abstain 1.4% (Strong Consensus)

#### Justification

A current retrospective study on 13,384 TKA and THA patients, who did not have increased VTE risk and had mechanical VTE prophylaxis together with early mobilization, showed that they had comparable VTE rates to those of patients who had pharmacological VTE prophylaxis [7].

Another RCT evaluated the effect of leg-elevated position in a total of 185 eligible patients undergoing coronary artery bypass grafting (CABG) who were randomly assigned to a supine group (*n* = 92) or a leg-elevation group (*n* = 93) [8]. Overall, DVT was detected in 25 patients (13.5%) [8]. There were more DVT cases in the supine-position group (17 patients, 18.4%) than in the leg-elevation group (eight patients, 8.6%), but this did not reach a significant difference [8].

Although bed rest had been recommended for treatment of acute DVT for a long time without strong supporting evidence, a meta-analysis of 13 studies in 3269 patients who had acute DVT and received conventional anticoagulation compared patient safety and pain between a bed-rest group and an early-ambulation group [9]. The early-ambulation group showed similar patient safety to the bed-rest group, in terms of incidence of new PE, progression of DVT, or DVT-related deaths [9]. Moreover, the early-ambulation group had better outcomes and lower remission of acute pain in the affected limb compared to the bed-rest group [9].

### 4A. Can active foot-ankle exercise be considered as mechanical VTE prophylaxis?

#### Recommendation

There is no evidence that foot-ankle exercise can prevent VTE. However, it does not cause harm to patients after limb surgery.

Delegate vote: Agree 87.7%, Disagree 1.3%, Abstain 11.0% (Strong Consensus)

### 4B. Can active breathing exercises be considered as nonpharmacological VTE prophylaxis?

#### Recommendation

There is no evidence that active breathing exercises can prevent VTE.

Delegate vote: Agree 97.2%, Disagree 1.4%, Abstain 1.4% (Strong Consensus)

#### Justification

There is no evidence to support the idea that foot-ankle exercise or active breathing exercises can prevent VTE in patients undergoing knee and hip arthroplasty and hip fracture surgery. However, we recommend that all patients practice these active exercise programs because there is no adverse effect.

A randomized controlled study showed that active ankle movement could reduce swelling of a patient’s leg after lower limb surgery and improve maximum venous outflow (MVO) and maximum venous capacity (MVC), which could prevent the formation of DVT after lower limb surgery [10]. A total of 174 patients were randomized as the intervention group (*n* = 96) and the control group (*n* = 78) [10]. The intervention group received routine nursing care and active ankle movement (30 times/min for 1–7 days after surgery) [10]. The results of the study revealed that thigh circumference in the intervention group significantly decreased compared with the control group on day 5, day 6, and day 7 after surgery, and crus circumference in the intervention group also significantly decreased compared with the control group on day 5, day 6, and day 7 [10]. The MVO and MVC in the intervention group significantly increased compared with the control group on the seventh day after surgery [10].

Deep breathing and active ankle exercise can increase blood flow velocity; however, there is no evidence that they are effective VTE prophylaxis methods. In the study of Kwon et al. [11], 20 healthy males (mean age 21.3 years) with no medical history of lower extremity disease were recruited for blood flow velocity testing in the femoral vein using a Doppler ultrasound. Among four different protocols, including quiet breathing while resting (QR), deep breathing (DB), ankle exercise with quiet breathing (AQB), and ankle exercising combined with deep breathing (ADB), there were statistically significant differences of venous peak blood flow velocity [11]. The mean (SD) peak blood flow velocity in the femoral vein was significantly different between each pair of the four protocols. The mean peak blood flow velocity in the femoral vein was highest with the ADB protocol, which implies that the ADB protocol may be useful in preventing blood stasis in patients at risk of DVT [11].

### 5. Is an inferior vena cava (IVC) filter recommended for PE prevention in knee and hip arthroplasty and hip fracture surgery patients who have a history of prior DVT?

#### Recommendation

No, IVC filter placement is not recommended for preventing PE in patients who have a history of prior DVT.

Delegate vote: Agree 95.9%, Disagree 1.4%, Abstain 2.7% (Strong Consensus)

#### Justification

Although a prospective study has shown that an IVC filter is safe and effective for prophylaxis against PE in high-risk patients who undergo joint arthroplasty [12], the IVC filter is recommended only in three clinical scenarios: 1) patients with documented VTE and classic indications, including absolute contraindication to anticoagulation, complication of anticoagulation resulting in cessation of therapy, and failure of anticoagulation; 2) patients with VTE and extended indications, including iliocaval DVT or extensive free-floating proximal DVT, difficulty establishing therapeutic anticoagulation, massive PE treated with thrombolysis or thrombectomy, chronic PE treated with thromboendarterectomy, thrombolysis for iliocaval DVT, VTE with limited cardiopulmonary reserve, recurrent PE with filter in place, poor compliance with anticoagulation, and high risk in complications of anticoagulation (e.g., risk for frequent falls); and 3) patients without VTE but with risk for developing VTE who cannot receive anticoagulation or be monitored for development of VTE, including trauma patients with high risk of VTE, surgical procedure in a patient at high risk for VTE, and medical condition with high risk of VTE [13].

### 6. Should a mechanical device for VTE prophylaxis be routinely applied in Asian patients undergoing knee and hip arthroplasty and hip fracture surgery?

#### Recommendation

Yes, a mechanical device for VTE prophylaxis should routinely be applied in all Asian patients undergoing knee and hip arthroplasty and hip fracture surgery.

Delegate vote: Agree 84.9%, Disagree 11.0%, Abstain 4.1% (Strong Consensus)

#### Justification

A retrospective study comparing Korean patients with and without application of IPCD after primary THA found a significantly lower incidence of symptomatic DVT in the patients with IPCD compared to the control group (0.1%, 1/870 cases, and 0.8%, 8/922 cases, respectively) [14]. Another study, evaluating symptomatic VTE after primary THA between patients with and without application of IPCD with low-dose aspirin for 6 weeks in both groups, found that the incidence of symptomatic VTE was lower in the IPCD group (1.3%) compared with the control group (4.1%). However, there was no statistical significance [15]. Although a retrospective study in Korean patients with no elevated VTE risk who were undergoing TKA reported that the use of IPCD alone did not reduce the DVT incidence [6], the recent retrospective study on 13,384 patients undergoing TKA and THA reported that mechanical prophylaxis with early mobilization provides comparable VTE rates to those for patients who had pharmacological VTE prophylaxis [7].

### 7. Is mechanical prophylaxis alone adequate for the prevention of VTE in Asian patients after knee and hip arthroplasty and hip fracture surgery?

#### Recommendation

It is inconclusive whether mechanical prophylaxis alone can be effective for VTE prevention in all Asian patients.

Delegate vote: Agree 95.9%, Disagree 2.7%, Abstain 1.4% (Strong Consensus)

#### Justification

Some studies from Asian countries support using mechanical devices alone for VTE prophylaxis in Asian patients undergoing knee and hip arthroplasty [14, 16–18]. A study on 741 patients who underwent 870 primary THAs with application of an IPCD found that three patients (0.3%) developed DVT (detected by sonography), one patient (0.1%) developed symptomatic DVT, and one patient (0.1%) developed symptomatic PE [14]. There were no reported fatal PEs [14]. The incidence of symptomatic DVT was significantly lower compared to that of the historical control group [7]. Although some studies provided a lower incidence of VTE in Asian patients undergoing total joint arthroplasty compared to their Western counterparts, VTE prevention results were similar between patients who had mechanical prophylaxis alone and patients who had combined mechanical and pharmacological prophylaxis [16, 17]. A retrospective comparative study enrolled 2798 patients who underwent TKA with mechanical VTE prophylaxis. It found that 102 of 2200 patients (4.6%), with no chemoprophylaxis, developed DVT compared to 32 of 540 patients (5.9%), with chemoprophylaxis, and the difference was not statistically significant [18]. The subgroup analysis found that 19 (0.8%) proximal DVTs and 83 (3.8%) distal DVTs developed in the patients without chemoprophylaxis, and 4 (0.7%) proximal DVTs and 28 (5.2%) distal DVTs developed in the patients with chemoprophylaxis [18]. The incidence of PE was equal in both groups, including 5 of 2200 patients (0.2%) without chemoprophylaxis and 1 of 540 patients (0.2%) with chemoprophylaxis [18]. A cohort study from Singapore on 966 patients who underwent TKA with routine mechanical prophylaxis without chemoprophylaxis found a similarly low prevalence of clinically significant VTE (0.82%) [19]. Seven patients developed DVT, and one patient died from a massive PE [19]. A retrospective study included 2891 consecutive TKAs in 1933 patients, in whom GCSs and IPCDs were used for VTE prophylaxis after TKA [20]. Fifty-three of 2891(1.83%) TKAs had suggestive symptoms or signs of VTE [20]; however only 26 (0.90%) were diagnosed as symptomatic VTE, including 10 (0.35%) symptomatic DVTs, 11 (0.38%) symptomatic PEs, 5 (0.17%) combined symptomatic DVTs and PEs, and 0 fatal PEs [20]. With appropriate patient selection and perioperative protocols, the investigators concluded that postoperative mechanical prophylaxis might be adequate for preventing VTE in Asians undergoing knee arthroplasty.

A prospective RCT by Cho et al. [21] studied the prevalence of total DVT in 148 East Asian patients undergoing TKA and compared a fondaparinux + GCS group (*n* = 74) and a placebo + GCS group (*n* = 74). The prevalence of total DVT was significantly higher in the placebo + GCS group (25.7%) than in the fondaparinux + GCS group (6.8%) [21]. There were no symptomatic VTEs in both groups at postoperative day 90. The authors concluded that, although combined mechanical and pharmacological prophylaxis was more effective in preventing total DVT, the prevalence of proximal DVT and PE was still low in East Asian patients [21]. They suggested that routinely combined mechanical and pharmacological prophylaxis should be reconsidered in regular East Asian patients, except in those with a high risk of VTE [21]. Similarly, another study by Kim et al. [5] of 1259 primary TKAs reported that the use of IPCD alone did not reduce the DVT incidence in Korean patients with low DVT incidence. Moreover, a prospective RCT by Woolson et al. [22] compared patients who underwent THA (total *N* = 217) among three groups: group A with IPCD alone (*N* = 76), group B with IPCD + aspirin (*N* = 72), and group C with IPCD + low-dose warfarin (*N* = 69). They then evaluated proximal DVT by venography or bilateral ultrasonography before discharge. The results showed no significant difference among the incidence of proximal DVT in all groups (group A 12%, group B 10%, and group C 9%) [22]. In a systematic review from Sobieraj et al., the authors compared the effectiveness of combined mechanical and pharmacological VTE prophylaxis and mechanical prophylaxis alone in patients who underwent TKA, THA, or hip fracture surgery [23]. The results showed that there were no significant differences in the risk of proximal DVT (risk ratio [RR] 0.78; 95% confidence interval [CI], 0.35–1.74) and PE (RR 1.57 [95% CI, 0.13–19.02]) between the groups [23].

By contrast, a study from Singapore reported that mechanical prophylaxis might not be adequate in reducing the rate of DVT after hip fracture surgery [24]. This study, on 454 patients who underwent hip fracture surgery with mechanical prophylaxis in all cases, showed an overall DVT incidence of 6.4% (29 patients) and a PE incidence of 1.3% (6 patients) [24]. Of 399 patients without chemoprophylaxis, 6.8% developed DVT (27 patients), 1% (4 patients) had a PE, and 0.25% (1 patient) had a PE without DVT, while of the 55 patients with chemoprophylaxis, 3.6% (2 patients) developed DVT, and 1.3% (1 patient) had a PE [24].

### 8A. When should the mechanical device for VTE prophylaxis be applied in patients undergoing knee and hip arthroplasty?

#### Recommendation

The mechanical VTE prophylactic device in patients undergoing knee and hip arthroplasty should be applied in the early postoperative period. The mechanical device for VTE prophylaxis can be applied intraoperatively, although there is no good evidence to support it.

Delegate vote: Agree 98.6%, Disagree 1.4%, Abstain 0% (Strong Consensus)

### 8B. When should the mechanical device for VTE prophylaxis be applied in patients undergoing hip fracture surgery?

#### Recommendation

The mechanical VTE prophylactic device in patients undergoing hip fracture surgery should be applied from the preoperative period.

Delegate vote: Agree 93.1%, Disagree 5.5%, Abstain 1.4% (Strong Consensus)

#### Justification

Wakabayashi et al. [25] reported the incidence of preoperative DVT by using Doppler ultrasound in patients who underwent primary and revision TKA and found asymptomatic DVT in 17.4% (56 of 322 patients), with increased risk in patients with revision TKA, rheumatoid arthritis, or connective tissue diseases. A high incidence of preoperative asymptomatic DVT encourages the use of mechanical prophylaxis as soon as possible, especially in some high-risk conditions [25].

Nam et al. reported a retrospective study of VTEs in 539 patients who underwent hip fracture surgery, by comparing the patients who received preoperative mechanical prophylaxis with IPCDs and GCSs from time of admission to surgery (135 patients) and the patients who did not receive preoperative mechanical prophylaxis before the operation (404 patients) [26]. All of the patients received postoperative mechanical and chemical prophylaxis until the day they were discharged from the hospital [26]. The study found the overall incidence of symptomatic DVT to be significantly lower in the group using preoperative and postoperative mechanical prophylaxis than in the group using only postoperative mechanical prophylaxis (2.2% vs. 7.4%) [26]. However, the incidence of symptomatic PE was not statistically different between both groups (1.5% vs. 3.7%). The study showed the effectiveness of using preoperative mechanical devices to prevent symptomatic DVT after hip fracture surgery [26].

### 9. What is the appropriate duration of mechanical VTE prophylaxis applied to patients?

#### Recommendation

Mechanical VTE prophylaxis should be used during hospitalization and extended after discharge until the patient’s independent ambulation is achieved.

Delegate vote: Agree 90.4%, Disagree 8.2%, Abstain 1.4% (Strong Consensus)

#### Justification

According to the 2018 National Institute for Health and Care Excellence (NICE) guidelines, in elective hip surgery, the duration of mechanical VTE prophylaxis (antiembolism stocking) should be applied until the patient is discharged, in combination with pharmacological prophylaxis for 28 days [27]. In elective knee surgery, they recommend the antiembolism stocking until discharge, combined with pharmacological prophylaxis for 14 days [27]. If a patient has a contraindication for pharmacological prophylaxis, one should consider an IPCD in elective knee replacement surgery until the patient is mobile and consider antiembolism stockings in elective hip replacement surgery until discharge [27].

In the RCT of Snyder et al., the authors compared the duration of mechanical VTE prophylaxis (IPCD during hospitalization only or extended use at home up to 6 weeks postoperatively) with aspirin for 3 weeks postoperatively after TKA [28]. The 6-week postoperative IPCD-therapy group experienced significantly superior DVT prophylaxis compared to the group receiving mechanical compression device therapy on an inpatient-only basis [28]. The DVT rate in the post-discharge IPCD-therapy group was 0%, and it was 23.1% for the inpatient IPCD group. There was also significantly higher satisfaction in the post-discharge IPCD therapy group. Thus, mechanical VTE prophylaxis can be extended to the period after the patient is discharged from the hospital [28].

### 10. Should mechanical VTE prophylaxis be indicated in all Asian patients who are contraindicated for pharmacological prophylaxis undergoing knee and hip arthroplasty or hip fracture surgery?

#### Recommendation

Yes, mechanical VTE prophylaxis is the most appropriate VTE prevention in Asian patients who are contraindicated for pharmacological prophylaxis. However, those patients who have acute thrombophlebitis, congestive heart failure, pulmonary edema, or limb ischemia due to peripheral vascular diseases should not receive mechanical VTE prophylaxis.

Delegate vote: Agree: 95.9%, Disagree: 2.7%, Abstain: 1.4% (Strong Consensus)

#### Justification

VTEs occur with an incidence ranging from 14 to 57 per 100,000 person-years [27, 29]. However, different countries appear to have different incidences of VTE following trauma and major orthopedic surgeries [27, 29]. Based on Asian VTE guidelines, mechanical prophylaxis using an IPCD is recommended as the primary method, and additional pharmacological prophylaxis is recommended if the thrombotic risk is high, e.g., because of advanced age, immobility, cancer, surgery, or trauma [29]. From the study of Sugano et al., mechanical thromboprophylaxis without anticoagulants was found to be useful in elective hip surgery in an Asian population [30]. There were no cases of fatal PE from the review of 3016 patients, and only five cases of symptomatic VTE were reported [30].

The 2018 NICE guidelines recommend mechanical VTE prophylaxis if pharmacological prophylaxis is contraindicated [27]. For fragility fractures of the hip and proximal femur, one should consider IPCDs at the time of admission [27]. For elective hip surgery, one should consider antiembolism stockings and continue until discharge from the hospital [27]. For elective knee replacement surgery, one should consider an IPCD and continue until the patient is mobile [27].

### 11. What are the proper size and length of the IPCD and GCS applied on the lower limb for VTE prophylaxis in knee and hip arthroplasty and hip fracture surgery?

#### Recommendation

The proper size and length of mechanical devices should correspond to the patient’s height and leg length. The length of mechanical devices should cover the lower limb, extending from the lower leg to the thigh. However, it is inconclusive whether a mechanical device covering the entire lower limb provides better VTE prevention than partial coverage.

Delegate vote: Agree 89.0%, Disagree 4.2%, Abstain 6.8% (Strong Consensus)

#### Justification

A mechanical VTE prophylactic device can prevent DVT formation by two mechanisms: decreasing venous stasis and activating fibrinolysis [31]. Its effect can be accomplished by compression of the foot or calf alone, or by sequential compression of either the foot and calf or the calf and thigh (14 studies; mostly TKA and THA, two spinal and one trauma patient) [3]. The Cochrane systematic review by Zhao et al. of 121 patients undergoing THA compared two types of IPCDs (calf-thigh compression and plantar compression). The review found no cases of symptomatic DVT or PE in either group in the first 3 weeks after surgery, but calf-thigh pneumatic compression was more effective in reducing thigh swelling than plantar compression [4].

An article on the Cochrane Database of Systematic Reviews studied the effectiveness of the length of a GCS (knee-length vs. thigh-length) and included three RCTs with 496 patients from various surgical specialties, including general, colorectal, hepatobiliary, gynecological, and ear, nose, and throat (ENT) surgery, urology, and neurosurgery [32]. There was no significant difference between varying lengths of a GCS in reducing the incidence of postoperative DVT [32]. Thus, the decision on which type of IPCD and GCS to use should rely on patient compliance, ease of use, and cost implication.

### 12. What is the optimal IPCD protocol for VTE prevention in Asian patients?

#### Recommendation

No specific IPCD protocol provides optimal VTE prevention. There are vastly different settings of pneumatic pressure, duration, and type of IPCD among different studies. However, an IPCD should be applied on both operated and nonoperated limbs.

Delegate vote: Agree 97.2%, Disagree 1.4%, Abstain 1.4% (Strong Consensus)

#### Justification

The 2012 American College of Chest Physicians (ACCP) guidelines and a comprehensive literature review recommend using IPCDs with 18 h of application time per day [33]. In the study of Delis et al. on compression pressure and range, it was found that coverage of the IPCD should include the foot and the calf at a frequency of two to four times per minute, with a pressure of 60–140 mmHg, to lower the venous pressure effectively [34]. According to the study of Giddings et al., a timing of IPCD of 2 h had a significant effect on enhancing fibrinolysis and suppressing procoagulant activation [35].

The 2018 NICE guideline for VTE prophylaxis makes the following recommendations [27].

For fragility fractures of the hip and proximal femur, one should consider intermittent pneumatic compression at the time of admission if pharmacological prophylaxis is contraindicated. This should continue until the patient no longer has significantly reduced mobility relative to his/her routine or anticipated mobility [27].

For patients undergoing elective hip replacement surgery, the guideline recommends low-molecular-weight heparin (LMWH) for 28 days combined with antiembolism stockings (until discharge) as one of the treatment options for VTE prophylaxis. One should also consider antiembolism stockings until discharge from hospital if pharmacological intervention is contraindicated [27].

For patients undergoing elective knee replacement surgery, one should consider intermittent pneumatic compression if pharmacological prophylaxis is contraindicated. This should be continued until the patient is mobile [27].

The American Academy of Orthopaedic Surgeons (AAOS) makes the following recommendations for prevention of symptomatic PE for elective hip and knee surgery [36]:

For Grade of Recommendation Moderate, it is suggested to use pharmacologic agents, mechanical compressive devices, or both for the prevention of venous thromboembolic disease for patients who are not at elevated risk beyond that of the surgery itself for VTE or bleeding [36].

For Grade of Recommendation Consensus, patients undergoing elective hip or knee arthroplasty and who have also had a previous venous thromboembolism, should receive pharmacological prophylaxis and mechanical compressive devices [36].

### 13. Do combined devices for mechanical VTE prophylaxis, such as IPCD and GCS, provide better effectiveness than a single device alone?

#### Recommendation

It is inconclusive whether combined devices for mechanical VTE prophylaxis will provide better effectiveness than a single device.

Delegate vote: Agree 94.5%, Disagree 1.4%, Abstain 4.1% (Strong Consensus)

#### Justification

Regarding mechanical VTE prophylaxis in hip and knee arthroplasty, both IPCDs and GCSs reduce the incidence of VTE by increasing venous blood flow, reducing venous distention, and preventing venous stasis [5, 28]. A prospective study by Fordyce and Ling [37] compared an A-V Impulse System foot pump + GCS and GCS alone in 84 patients who had undergone THA. The incidence of postoperative DVT was significantly higher in the patients using GCS alone (40% vs. 5%) [37]. However, the retrospective study in elective primary TKA by Kim et al. [5] compared an IPCD + GCS group (425 patients) vs. a group using GCS alone (420 patients). The results showed that the overall DVT was not significantly different between the two groups (14.8% in GCS alone, 11.3% in IPCD + GCS) [5]. The incidence of symptomatic DVT was 0.7% in both groups, with no fatal PE observed [5]. Therefore, it remains controversial whether combined devices have better effectiveness than a single device.

In hip fracture surgery, mechanical VTE prophylactic methods can reduce the incidence of VTE [38]. In a study by Mehta et al., the authors showed that using an IPCD for ≥20 h per day before and after surgery could reduce VTE in 434 hip fracture patients. The incidence of DVT was 11 (2.5%) and that of PE was 2 (0.5%) [38], compared with a previous study, which showed an incidence of DVT of 8% in 104 elderly hip fractures without mechanical thromboprophylaxis [39]. However, there are inadequate data for fatal PE and mortality rates to make a conclusion. No evidence-based study compares the effectiveness between combined devices and a single device for mechanical VTE prophylaxis [40].

### 14. Do variations of IPCDs (such as respiratory-synchronized, mobile IPCDs) available in the market provide different efficacies on VTE prophylaxis?

#### Recommendation

Inconclusive, there is no evidence whether different modes/types of IPCD provide different efficacies on VTE prophylaxis. There is only sparse evidence investigating the effects of various modes of IPCD on VTE prophylaxis.

Delegate vote: Agree 97.2%, Disagree 1.4%, Abstain 1.4% (Strong Consensus)

#### Justification

In general, IPCDs can be categorized into single-chamber or multichamber, constant-pressure or sequential-pressure, and slow gradual or rapid-inflation devices, as well as portable or nonportable devices [3]. An IPCD is appropriate for VTE prophylaxis when used in the setting of current clinical guidelines [29]. However, a systematic review by Pavon et al. showed the limitations of several evidence-based studies in comparing the effectiveness of each type of device [3]. A portable IPCD has the advantage of continued use during ambulation in the early postoperative period [41].

A multicenter study by Colwell et al. [42] showed noninferior effectiveness of the use of a mobile compression device alone in preventing VTE when compared to use of a mobile compression device and pharmacological prophylaxis. The study by Froimson el al [41]. showed that a mobile IPCD proved significantly more effective than a standard IPCD when used in conjunction with LMWH for DVT prevention in high-risk orthopedic patients. The results showed that the mobile IPCD had lower rates of DVT (1.3% vs. 3.6%), lower rates of symptomatic PE (0% vs. 0.66%), better compliance (83% vs. 49%), and shorter length of hospital stay (4.2 vs. 5.0 days) [41]. However, Arsoy et al. [43], in a retrospective study, compared two types of IPCDs (nonmobile + LMWH 14 days vs. mobile + aspirin once daily 14 days), and the results were not different in the rate of symptomatic VTEs between both groups (THA: 2.6% for the nonmobile group vs. 1.9% for the mobile group; *p* = 1.0; TKA: 1.1% vs. 0%, respectively). This study showed the beneficial effects of both mobile and nonmobile IPCDs [43].

Regarding the mode of IPCD, an RCT by Koo et al. [44] compared an IPCD with alternate sequential compression (ASCD) vs. a simultaneous sequential compression device (SSCD) of both legs in 34 patients who underwent knee and spine surgery. The outcomes found no significant difference in asymptomatic distal DVT (11.8% in the ASCD group vs. 29.4% in the SSCD group, *p* = 0.331) [44]. There were no occurrences of symptomatic DVT or proximal DVT in either group [44]. Another RCT study compared two different methods of IPCD (simultaneous compression with fixed cycling rate [SF] vs. alternate compression with adjusted cycling rate [AA]) in 54 patients undergoing TKA [45]. The results found no significant difference in total DVT (55.6% in the AA group vs. 51.9% in the SF group), although the SF group showed better hemodynamic parameters [45]. A meta-analysis by Elbuluk et al. [46] compared the effectiveness of respiratory-synchronized compression devices (RSCDs) and nonsynchronized intermittent pneumatic compression devices (NSIPCDs) to pharmacological prophylaxis for preventing VTE after total joint arthroplasty. The results showed that both devices had effectiveness in preventing VTE. In the RSCD group, the RRs of DVT and PE were 0.47 (95% CI, 0.27–0.80; *I*^2^ = 0%) and 0.62 (95% CI, 0.29–1.32; *I*^2^ = 0%), respectively [46]. In the NSIPCD group, the RRs of DVT and PE were 0.51 (95% CI, 0.39–0.67; *I*^2^ = 69%) and 0.24 (95% CI, 0.04–1.47; *I*^2^ = 0%), respectively [46].

## Data Availability

Not applicable.
